# Comparison of different anesthesia modalities during percutaneous kyphoplasty of osteoporotic vertebral compression fractures

**DOI:** 10.1038/s41598-021-90621-9

**Published:** 2021-05-27

**Authors:** Chaoyuan Ge, Xucai Wu, Zijun Gao, Zhengwei Xu, Dingjun Hao, Liang Dong

**Affiliations:** 1 Department of Spine Surgery, Hong-Hui Hospital, Xi’an Jiaotong University College of Medicine, Xi’an, 710054 China; 2 Department of Anesthesiology, Hong-Hui Hospital, Xi’an Jiaotong University College of Medicine, Xi’an, 710054 China

**Keywords:** Fracture repair, Medical research, Drug development

## Abstract

Local and general anesthesia are the main techniques used during percutaneous kyphoplasty (PKP); however, both are associated with adverse reactions. Monitored anesthesia with dexmedetomidine may be the appropriate sedative and analgesic technique. Few studies have compared monitored anesthesia with other anesthesia modalities during PKP. Our aim was to determine whether monitored anesthesia is an effective alternative anesthetic approach for PKP. One hundred sixty-five patients undergoing PKP for osteoporotic vertebral compression fractures (OVCFs) were recruited from a single center in this prospective, non-randomized controlled study. PKP was performed under local anesthesia with ropivacaine (n = 55), monitored anesthesia with dexmedetomidine (n = 55), and general anesthesia with sufentanil/propofol/sevoflurane (n = 55). Perioperative pain was assessed using a visual analogue score (VAS). Hemodynamic variables, operative time, adverse effects, and perioperative satisfaction were recorded. The mean arterial pressure (MAP), heart rate, VAS, and operative time during monitored anesthesia were significantly lower than local anesthesia. Compared with general anesthesia, monitored anesthesia led to less adverse anesthetic effects. Monitored anesthesia had the highest perioperative satisfaction and the lowest VAS 2 h postoperatively; however, the monitored anesthesia group had the lowest MAP and heart rate 2 h postoperatively. Based on better sedation and analgesia, monitored anesthesia with dexmedetomidine achieved better patient cooperation, a shorter operative time, and lower adverse events during PKP; however, the MAP and heart rate in the monitored anesthesia group should be closely observed after surgery.

## Introduction

Percutaneous kyphoplasty (PKP) is a safe and effective minimally invasive surgical treatment for osteoporotic vertebral compression fractures (OVCFs)^1,2^. Because of the rapid onset, precision, and cost-effectiveness, local anesthesia has been widely used during PKP. Nevertheless, local anesthetics cannot be injected into the vertebral body, thus, some patients experience severe pain that can even be intolerable, especially during PKP, which is more complex than percutaneous vertebroplasty^3^. General anesthesia is an important medical application that is used during PKP to provide a comfortable surgical condition for both patients and physicians; however, general anesthesia is likely to increase the risk of anesthetic adverse effects, hospital stay, and costs^4^. In recent years, dexmedetomidine, a highly selective α^2^ adrenergic agonist, has gradually been adopted for clinical therapy. Monitored anesthesia with dexmedetomidine delivers intraoperative sedation and analgesia, thus enabling intraoperative nerve injury assessment through intraoperative painful arousal, and rarely cause respiratory depression^5^. Monitored anesthesia with dexmedetomidine has rarely been used during PKP, and no study comparing monitored anesthesia with local and general anesthesia has been reported. In view of the known disadvantages with local and general anesthesia, we determined if monitored anesthesia is an effective alternative anesthetic approach during PKP. Therefore, a prospective, non-randomized controlled trial was conducted to compare the effects of the three different anesthesia modalities during PKP.

## Materials and methods

A prospective, non-randomized controlled study was conducted. All patients underwent PKP for a single-level OVCF from August 2015 to November 2019. The patients were free to choose the anesthetic method based on their preference. All patients were provided written informed consent. The study was approved by the Human Research Ethics Committee at Honghui Hospital (201506001). Clinical trial registration: ChiCTR2100046162, 1st August 2015. Written informed consent was obtained from all patients. The methods were carried out in accordance with the relevant guidelines and regulations. Demographic data were shown in Table 1.

**Table 1:** Baseline demographic.

Parameters	Local anesthesia	Monitored anesthesia	General anesthesia	P value
Number of cases	55	55	55	
Average age, years	76.8 ± 7.2	75.4 ± 6.9	75.7 ± 5.5	0.503
**Age range, years**				
Minimum	64	61	64	
Maximum	96	95	91	
Sex, male:female	20:35	19:36	17:38	0.828
Body Mass Index, kg/m^2^	21.6 ± 3.4	22.2 ± 2.8	21.9 ± 2.5	0.505
**Body Mass Index range, kg/m**^**2**^				
Minimum	16.7	18.9	18.4	
Maximum	30.5	29.6	30.4	
Height (cm)	169.3 ± 8.1	169.8 ± 6.9	167.8 ± 6.9	0.361

P ≥ 0.05 indicated no statistical difference.

The inclusion criteria were as follows: (1) a single-level OVCF was diagnosed by MRI and bone density (T score <  − 2.5); (2) severe back pain associated with an OVCF, and unresponsive to analgesic medication for at least 2 weeks; and (3) the presence of a ≥ 15% height loss of the fractured vertebra^6^. The exclusion criteria were as follows: (1) compression of the spinal cord and nerve roots; (2) inability to cooperate, such as Alzheimer’s disease and other forms of dementia; and (3) pathologic fractures, such as vertebral metastatic cancer or osteomyelitis.

### Anesthesia method

The patients were divided into three groups based on the anesthetic method: local anesthesia group; monitored anesthesia group; and general anesthesia group. The detailed methods were as follows:

Oxygen was delivered at 3  L/min by conventional mask; and electrocardiographic data, pulse oxygen saturation, respiratory rate, heart rate, and blood pressure were monitored.

#### Local anesthesia group

The skin and subcutaneous tissue at the insertion site was infiltrated with 3–5 mL of 0.4% ropivacaine.

#### Monitored anesthesia group

In addition to the above local anesthesia procedure, patients received an intravenous injection of 5 mg of dezocine based on weight and age. Subsequently, anesthesia was induced by 0.5 μg/kg of intravenous dexmedetomidine as a loading dose, and maintained thereafter via a continuous intravenous infusion of dexmedetomidine (1 μg/kg/h).

#### General anesthesia group

Anesthesia was induced with 0.2 μg/kg of intravenous sufentanil, 1 mg/kg of intravenous propofol, and 0.2 mg/kg of intravenous cisatracurium to facilitate endotracheal intubation, then anesthetized by inhalation of 1% sevoflurane. The maintenance of anesthesia was achieved with remifentanil (0.15 μg/kg/min). Ten minutes before the estimated end of surgery, 5 μg of sufentanil was administered intravenously.

### Surgical procedure

All PKP procedures were performed by experienced surgeons, as described in a previous study (KMC; Kinetic Medical Co., LTD, Shanghai, China)^7^. The patient assumed the prone position. A unilateral transverse process-pedicle approach was adopted to perform all PKPs. The needle puncture through the pedicle and into the anterior one-third of the vertebral body was performed under fluoroscopy. Then, a balloon was inserted into the vertebral body and expanded. Finally, bone cement was injected into the vertebral body under lateral fluoroscopy guidance. Intraoperative nerve injury was evaluated by motor evoked potentials (MEPs) in the general anesthesia group.

### Outcome evaluation

The mean arterial pressure (MAP), heart rate, and visual analogue score (VAS) were recorded at the following times: 1 h before anesthesia (time point 1); trocar puncture into the vertebral body (time point 2); injection of bone cement into the vertebral body (time point 3); 2 h postoperatively (time point 4); and 1 day postoperatively (time point 5). The operative time, bone cement leakage, neurologic injuries, adverse anesthetic reactions, and perioperative satisfaction were assessed.

The operative time was measured from the time of needle puncture to wound closure. Postoperative neurologic injuries were assessed based on symptoms and MEPs. Adverse anesthetic reactions were defined as vomiting, drowsiness, hypotension (a MAP < 60 mmHg), bradycardia (heart rate < 60/min), hypoxemia, and pharyngalgia. Perioperative satisfaction was assessed using a 5-point Likert scale at the time of hospital discharge^8^.

### Statistical analysis

A blinded method was adopted to analyze the experimental data. SPSS statistical software (version 19.0; SPSS, Inc., Chicago, IL, USA) was used. A repeated measures ANOVA was used in three group comparisons with repeated measurement data. One-way ANOVA was used in three group comparisons with univariate data. Enumeration data were evaluated using a chi-square test or Fisher’s exact test. A P value < 0.05 was considered statistically significant.

## Results

One hundred sixty-five patients who underwent PKP were included in the study; the number of patients in each group was 55. There were no changes in the anesthetic plan for any patient; the data collection was completed in all patients. There were no significant differences in demographic data between the three groups (Table 1).

### MAP and heart rate

**Figure 1:**
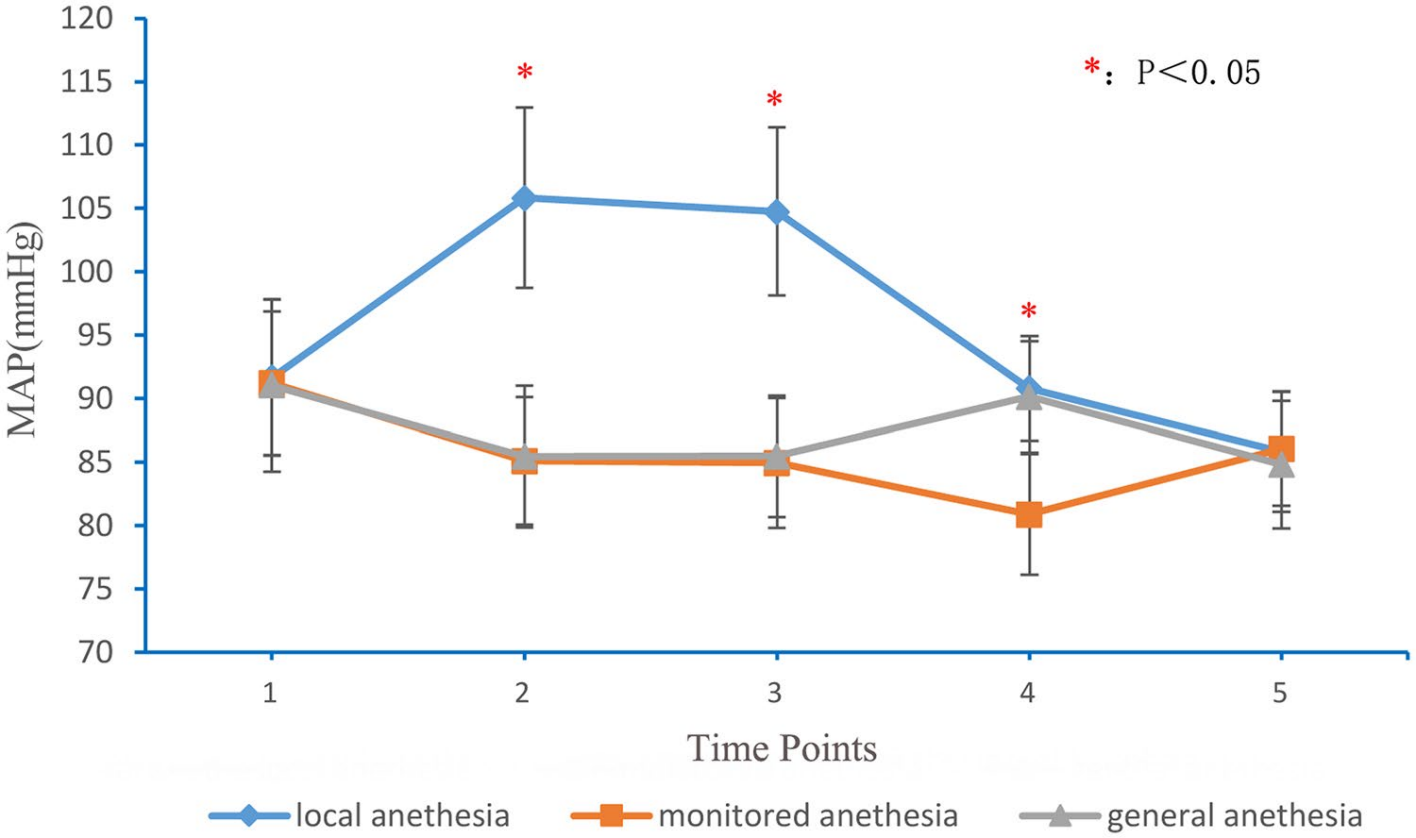
MAP at different time points for local anesthesia, monitored anesthesia and general anesthesia groups. *MAP* mean arterial pressure. 1: 1 h before anesthesia; 2: puncture of trocar into vertebral body; 3: injection of bone cement into vertebral body; 4: 2 h after operation; 5: 1 day after operation.

**Figure 2:**
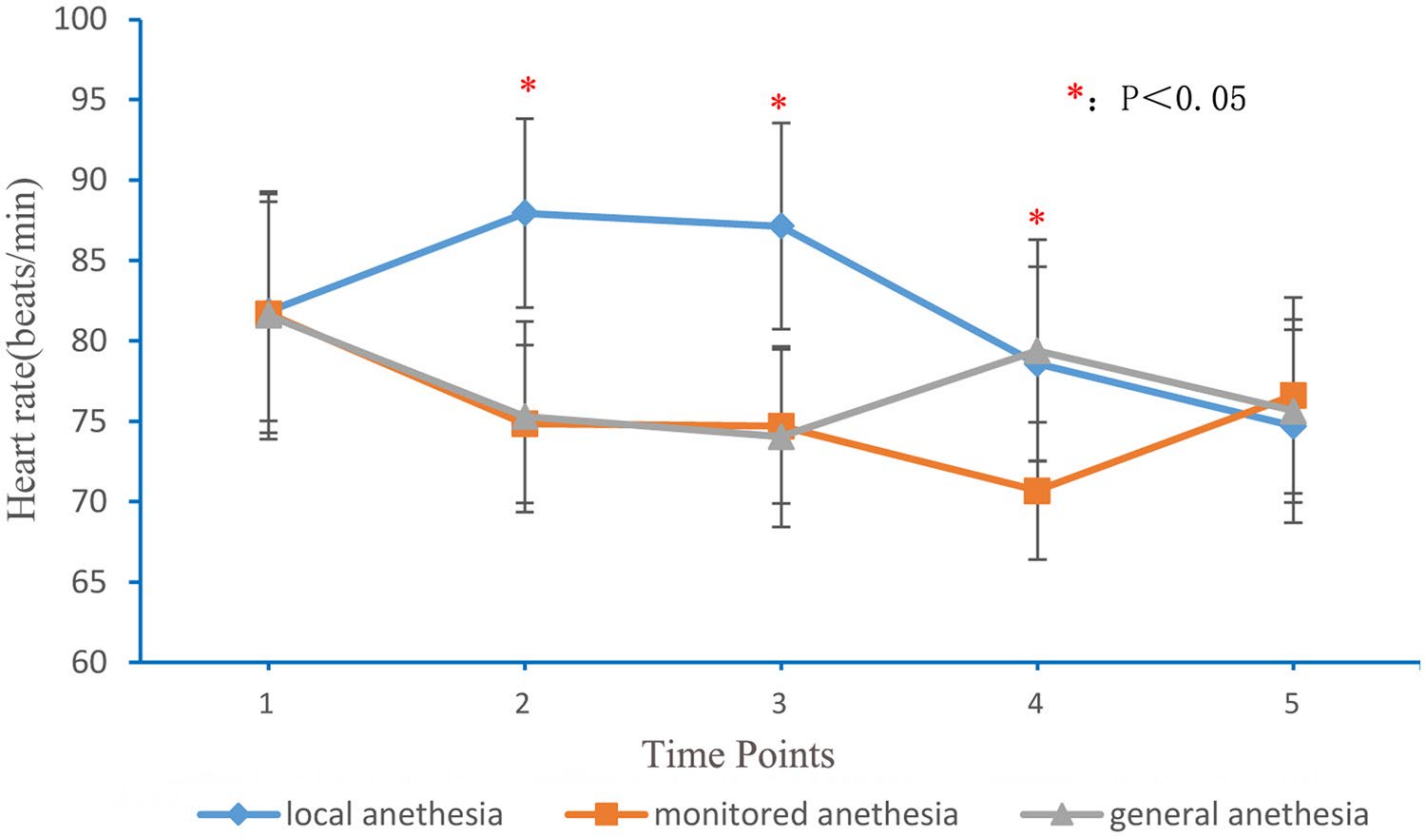
Heart rate at different time points for local anesthesia, monitored anesthesia and general anesthesia groups. 1: 1 h before anethesia; 2: puncture of trocar into vertebral body; 3: injection of bone cement into vertebral body; 4: 2 h after operation; 5: 1 day after operation.

The MAP and heart rate are shown in Figs. 1 and 2. No statistical differences existed between the three groups with respect to MAP and heart rate 1 h before anesthesia and 1 day postoperatively. Analysis of variance showed that these differences in the MAP and heart rate were statistically significant at the time of trocar puncture into the vertebral body, injection of bone cement into the vertebral body and 2 h postoperatively. The local anesthesia group had the highest MAP and heart rate at the time of trocar puncture into the vertebral body, injection of bone cement into the vertebral body, and 2 h postoperatively. There were no statistically significant differences in the MAP and heart rate between the monitored and general anesthesia groups at the time of trocar puncture into the vertebral body and injection of bone cement into the vertebral body. The monitored anesthesia group had a significantly lower MAP and heart rate than the general anesthesia group 2 h postoperatively (P < 0.01); however, hypotension and bradycardia were not observed in the monitored anesthesia group.

### Visual analogue score

The VAS data are listed in Table 2. There were no statistically significant differences between the groups with respect to VAS 1 h before anesthesia and 1 day postoperatively. The intraoperative VAS could not be assessed in the general anesthesia group. The VAS was significantly lower in the monitored anesthesia group than the local anesthesia group at the time of trocar puncture into the vertebral body and injection of bone cement into the vertebral body (P < 0.01). The monitored anesthesia group had the lowest VAS 2 h postoperatively; however, no statistically significant differences in VAS were observed between the other two groups 2 h postoperatively.

**Table 2:** Visual analogue score (x ± s).

Group	n	1	2	3	4	5
Local anesthesia	55	6.6 ± 1.6	7.2 ± 1.1^#^	6.9 ± 0.9^#^	3.1 ± 1.0	1.7 ± 0.7
Monitored anesthesia	55	6.6 ± 1.6	2.0 ± 0.9	1.8 ± 0.7	1.7 ± 0.7^#^	1.8 ± 0.8
General anesthesia	55	6.5 ± 1.6	0	0	2.6 ± 0.8	1.6 ± 0.7
F		0.062	645.001	1122.971	37.658	0.823
P		0.940	0.000	0.000	0.000	0.442

^#^Statistical difference among three groups (P < 0.05). Repeated Measures Anova was used in three or two group comparisons. 1: 1 h before anesthesia; 2: puncture of trocar into vertebral body; 3: injection of bone cement into vertebral body; 4: 2 h after operation; 5: 1 day after operation. F: F test value.

### Operative time, cement leakage, nerve injuries, adverse effects during anesthesia, and perioperative satisfaction

The operative time, cement leakage, nerve injuries, adverse effects during anesthesia, and perioperative satisfaction data are listed in Tables 3 and 4. The local anesthesia group had the longest operative times and the lowest perioperative satisfaction among the three groups. Nevertheless, the local anesthesia group had the lowest adverse anesthetic reactions. Perioperative satisfaction was highest in the monitored anesthesia group. There were no statistically significant differences in the operative times between the monitored and general anesthesia groups. The differences in intraoperative nerve injuries and the rate of cement leakage were not statistically different among the three groups.

**Table 3:** Operating time, the rate of cement leakage, neurological damage.

Group	n	Operating time	Cement leakage (n)	Neurological damage (n)	Intraoperative satisfaction
Local anesthesia	55	39.6 ± 7.0^#^	9	0	2.6 ± 0.9
Monitored anesthesia	55	33.5 ± 5.0	4	1	3.6 ± 0.8^#^
General anesthesia	55	32.4 ± 4.9	3	1	2.8 ± 0.7
F		25.245	4.291 (χ^2^)	1.254	25.140
P		0.000	0.117	1.000	0.000

^#^Statistical difference among three groups (P < 0.05). One-way ANOVA was used in three group comparisons with univariate data. Enumeration data (cement leakage) was compared using chi-square test; Enumeration data (neurological damage) was compared using Fisher’s exact test. F: F test value.

**Table 4:** Adverse effects during anesthesia (n).

Group	n	Vomiting	Drowsiness	Hypotension	Bradycardia	Hypoxemia	Pharyngalgia	Total adverse effects rate (%)
Local anesthesia	55	2	0	1	1	2	0	1.82
Monitored anesthesia	55	5	22	5	4	3	0	11.82
General anesthesia	55	16	25	2	2	8	21	22.42^#^

^#^Statistical difference among three groups (P < 0.05).

## Discussion

OVCFs usually occur in elderly patients with osteoporosis. The mean age of the patients in our study was 76 years. Severe dynamic pain caused by an OVCF affects the quality of life, and can even lead to long-term bed rest among these patients, which then increases the risk of pneumonia, thromboses, and bedsores. Moreover, some patients are admitted to the hospital with Kummell disease, further causing vertebral collapse and increasing the difficulty of treatment^9,10^. A PKP provides early, effective vertebral stabilization and pain relief among elderly patients^11^. In the current study, the VAS scores decreased from 6.6 preoperatively to 1.7 one day postoperatively. Apan et al.^12^ recently reported that segmental epidural anesthesia offered better advantages than general anesthesia in terms of postoperative analgesia, analgesic consumption, and early recovery; however, patients must maintain a lateral and flexed position during administration of epidural anesthesia, which may aggravate a vertebral fracture. In addition, epidural anesthetics may infiltrate into the subarachnoid space and cause total spinal anesthesia or respiratory inhibition. Dexmedetomidine has been effectively used in minimally invasive surgeries^13^. The results in the current study are consistent with the literature; dexmedetomidine was shown to provide effective sedation and analgesia without affecting communication between the physician and patient. Detailed discussions are presented as follows.

### Application of local anesthesia

Injection of lidocaine for local anesthesia has been widely used during PKP^14,15^. Low-dose ropivacaine induces a conduction block of sensory function without motor dysfunction^5^. Therefore, we applied 0.4% ropivacaine for local anesthesia during PKP. Compared with the other two groups, local anesthesia in this study had the lowest adverse anesthetic reactions. Local anesthesia also effectively prevented intraoperative neurologic injuries by performing the PKP in the awake state. Because local anesthetics cannot be injected into the vertebral body, the VAS in the local anesthesia group was highest during trocar insertion and bone cement injection, which caused the highest intraoperative MAP and heart rate among the three groups. Unbearable intraoperative pain caused poor patient cooperation, and further led to the longest operative time. Injection of lidocaine into the intravertebral body is contraindicated because local anesthetics may be disseminated to the entire body via the vertebral vessels, thus, causing respiratory inhibition or death^12^. No differences were observed between the groups with respect to cement leakage. Indeed, it is likely that cement leakage is most often associated with the type of vertebral fracture.

### Application of general anesthesia

General anesthesia has been widely used for open surgeries. General anesthesia is not utilized in most PKPs because of the longer preoperative preparation time and higher hospitalization costs^3,4^. Our study showed that the general anesthesia group had the highest rate of adverse anesthetic effects. Of the patients who received general anesthesia, 29.1% had postoperative vomiting and 38.2% had pharyngalgia secondary to the intubation. The nerve injury rate in the general anesthesia group was not statistically different compared to the other two groups due to MEP monitoring, but MEP increases the economic burden for the patients. Compared to local anesthesia, general anesthesia can maintain a more stable MAP and heart rate. Surgeons could also perform the surgery in less time. Some studies have reported that PKP without MEP monitoring might be performed under general anesthesia by more experienced surgeons^3,12^.

### Application of monitored anesthesia

Dexmedetomidine produces hypnosis by eliminating the inhibitory effect of the locus coeruleus on basal forebrain γ -amino butyric acid, and sedation caused by dexmedetomidine is similar to the sedation caused by natural sleep, from which a patient is easily aroused during monitored anesthesia with dexmedetomidine^16^. As an opioid receptor agonist, dezocine provides analgesia without respiratory depression. Dezocine is commonly used to enhance the analgesic effect of dexmedetomidine.

Patients had the highest perioperative satisfaction with monitored anesthesia because monitored anesthesia had a significantly lower intensity of pain sensation compared to local anesthesia and fewer adverse anesthetic reactions compared to general anesthetic. Based on better patient cooperation during trocar insertion and bone cement injection, monitored anesthesia had the shortest operative time compared to local anesthesia. Moreover, severe pain can easily awaken a patient when a nerve is touched, which could effectively prevent intraoperative neurologic injury.

Because of the continuous postoperative analgesic and sedative effects, the VAS in the monitored anesthesia group was the lowest 2 h postoperatively; however, the central and peripheral sympatholytic effects of dexmedetomidine may lead to bradycardia and hypotension^17,18^. In the current study, the MAP and heart rate in the monitored anesthesia group were significantly lower than the local and general anesthesia groups 2 h postoperatively. Tanriverdi et al.^19^ suggested that low doses of dexmedetomidine (loading dose, 1 μg/kg; continuous dose, 0.7 μg/kg/h) might reduce the rate of hypotension and bradycardia^19^, which was in agreement with our findings.

### Limitations of the study

The limitations of the current study were as follows: (1) non-random allocation may contribute to selection bias; (2) a lack of blinding in patients and surgeons may lead to performance bias; (3) dexmedetomidine may induce the risk of hypotension and bradycardia, thus, more patients are needed to analyze the side effects of dexmedetomidine; and (4) the radiation dose and injection volume of bone cement among the three anesthesia groups should be compared in future studies.

## Conclusions

Compared to local anesthesia, monitored anesthesia achieved better intraoperative sedation and analgesia, and had a shorter operative time. Compared to general anesthesia, monitored anesthesia led to fewer adverse anesthetic effects. In addition, monitored anesthesia resulted in better perioperative satisfaction than the other two anesthetic methods. Therefore, monitored anesthesia might be a suitable alternative for PKP.
